# Integrative single-cell and bulk transcriptomics with reverse network pharmacology prioritizes macrophage-associated candidate genes in Lyme arthritis

**DOI:** 10.3389/fimmu.2026.1938154

**Published:** 2026-10-13

**Authors:** Yan Dong, Yanshuang Luo, Yantong Chen, Meng Liu, Yingchen Zhou, Yutian Yang, Xuesong Chen, Fusong Yang, Zhi Liang, Liye Zi, Hongbing Gao, Zhichao Cao, Fucun Gao, Chao Song, Guozhong Zhou

**Affiliations:** 1 Department of Pain Medicine, The Affiliated Anning First People’s Hospital of Kunming University of Science and Technology, Kunming, Yunnan, China; 2 School of Basic Medical Sciences, Kunming University of Science and Technology, Kunming, Yunnan, China; 3 Faculty of Life Science and Technology and the Affiliated Anning First People’s Hospital, Kunming University of Science and Technology, Kunming, Yunnan, China; 4 Clinical Medical College, Kunming University, Kunming, Yunnan, China; 5 First School of Clinical Medicine, Kunming Medical University, Kunming, Yunnan, China

**Keywords:** Lyme arthritis, macrophage, MSR1, network pharmacology, single-cell RNA sequencing

## Abstract

**Introduction:**

Lyme arthritis (LA) involves complex immune dysregulation, and macrophages contribute centrally to persistent joint inflammation. However, synovial macrophage heterogeneity and its potential relevance to therapeutic intervention remain incompletely defined.

**Methods:**

We integrated scRNA-seq data from *Borrelia burgdorferi* (*Bb*)-infected murine joints (GSE233850) with bulk RNA-seq data (GSE283617). The workflow included Seurat clustering, Monocle 3 trajectory inference, CellChat communication analysis, and functional enrichment. We constructed a PPI network from intersecting genes and applied five complementary topological measures to prioritize consensus hubs. Candidate gene expression was evaluated in an external transcriptomic dataset (GSE63085) and by qRT-PCR. CTD-based association analysis and molecular docking were used to prioritize candidate compounds. Virtual knockout analysis explored the potential regulatory effects of hub genes.

**Results:**

The two pooled libraries differed descriptively in the relative representation of nine major cell populations. Among macrophages/monocytes, we resolved seven marker-defined states. Monocle 3 trajectories were rooted at Classical_Inflam_Mono, and CellChat highlighted Tgfb1-(Tgfbr1+Tgfbr2) as a prominent inferred TGF-β axis. Intersecting GSE283617 with GSE233850 yielded 121 shared genes, and GSEA showed negative enrichment of oxidative phosphorylation during infection. Mrc1 and Msr1 were the only genes ranked among the top 10 by all five measures: Degree, Bottleneck, EcCentricity, MNC, and MCC. Across these complementary analyses, Msr1 emerged as a candidate *Bb*-responsive gene potentially relevant to LA. A CTD-based source-compound-gene network and molecular docking prioritized curcumin and demethoxycurcumin for future experimental testing.

**Conclusion:**

These findings provide an exploratory atlas of macrophage transcriptional states in the analyzed LA model and identify Msr1 as a candidate infection-responsive gene. Its disease specificity and clinical utility require validation in joint-derived specimens from patients with clinically confirmed LA. The computationally prioritized compounds remain hypotheses that require direct binding and functional testing.

## 1 Introduction

Lyme arthritis (LA), the most prevalent late-stage manifestation of Lyme disease caused by the tick-borne spirochete *Bb*, remains a substantial therapeutic challenge. Although antibiotics are generally effective, approximately 10–20% of patients develop antibiotic-refractory LA, with joint inflammation persisting for months or years despite spirochetal clearance (1–3). This dissociation between inflammation and viable infection suggests dysregulated host immune memory or impaired inflammatory resolution (4–7). Defining the cellular drivers of this persistent “sterile inflammation” is therefore important for developing host-directed therapies beyond antibiotics.

Macrophages have a central role in LA pathogenesis (8, 9). Following *Bb* infection, they secrete the chemokines CCL4 and CCL2, recruit monocytes and T cells, and amplify inflammation through phagocytosis and antigen presentation (10, 11). Animal and human studies further implicate macrophages and pro-inflammatory cytokines, including IL-1β, TNF-α, and IFN-γ, in maintaining joint inflammation (12–14). However, macrophages also contribute to *Bb* clearance and inflammatory resolution (15). Their functions depend on activation state, cytokine context, and immune-cell interactions, underscoring the need to resolve macrophage heterogeneity in LA.

Single-cell RNA sequencing (scRNA-seq) can resolve tissue heterogeneity and cell-specific transcriptional programs (16–18). However, few studies have integrated scRNA-seq with bulk RNA sequencing to examine macrophage responses in LA. Connecting high-resolution transcriptional signatures with experimentally testable therapeutic hypotheses may also support translational investigation.

We integrated scRNA-seq data with a publicly available bone marrow-derived macrophage transcriptome to characterize host immune responses to *Bb* infection. This analysis prioritized MSR1 as a candidate for further evaluation. We then used CTD-derived associations and molecular docking to generate testable hypotheses for candidate natural compounds.

## 2 Methods

We integrated the scRNA-seq dataset GSE233850 and the bulk RNA-seq dataset GSE283617 from the Gene Expression Omnibus (GEO) to investigate macrophage/monocyte transcriptional states and candidate infection-responsive genes in LA. As shown in **Figure 1**, the workflow comprised eight stages. These were (1) data acquisition and preprocessing; (2) scRNA-seq clustering, trajectory inference, and cell-cell communication profiling; and (3) functional annotation using GSVA, KEGG, and GO analyses. Subsequent stages included (4) PPI network construction and consensus topological prioritization; and (5) parallel expression comparisons and exploratory ROC analyses of Mrc1 and Msr1. We then performed (6) qRT-PCR assessment; (7) in silico virtual knockout analysis of Msr1; and (8) CTD-based compound mapping and molecular docking.

**Figure 1:**
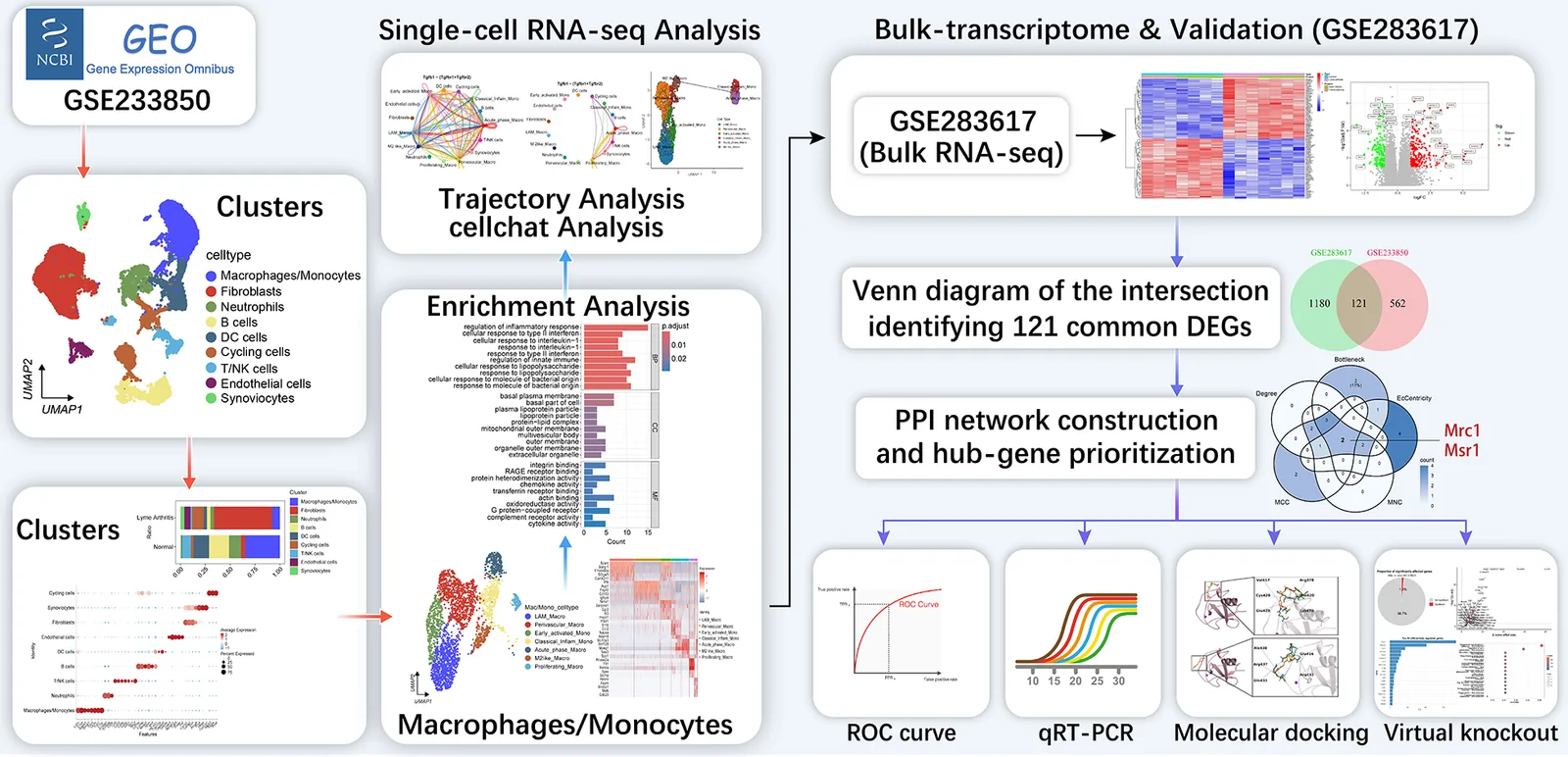
Comprehensive workflow diagram.

### 2.1 Single-cell RNA sequencing data quality control and processing

We retrieved scRNA-seq data from GEO dataset GSE233850, derived from the study “Single-cell RNA sequencing of murine ankle joints over time reveals distinct transcriptional changes following *Borrelia burgdorferi* infection” (19). We selected ankle-joint samples from uninfected controls and mice at 2 weeks post-infection. This time point represents peak pathological inflammation and transcriptional remodeling across major cell populations, including macrophages and fibroblasts. It therefore provided a window for examining acute innate-adaptive immune crosstalk before resolution. For each condition, ankle tissues from 10 mice were pooled into a single scRNA-seq library. The primary comparison therefore comprised one pooled control library and one pooled 2-week post-infection library (n = 1 biological library per condition). Cell-level observations were not treated as independent biological replicates (20). Quality-control criteria were more than 500 expressed genes per cell, expression of each gene in at least 3 cells, and mitochondrial RNA content below 20% (21). Data were processed using Seurat. NormalizeData was applied with a scale factor of 10,000, followed by selection of 2,000 highly variable genes using FindVariableFeatures. Data were scaled using ScaleData, reduced using RunPCA, and corrected for batch effects using RunHarmony with group.by.vars = “orig.ident”. After quality control, 16,841 cells were retained for analysis (22).

### 2.2 Dimensionality reduction and data clustering

We first performed principal component analysis on highly variable genes using RunPCA (23, 24). Data were then integrated with RunHarmony using 2,000 anchor points. Clusters were identified using FindNeighbors and FindClusters. Finally, RunUMAP was applied with dimensions 1:14 and a resolution of 0.5 to visualize the clusters.

### 2.3 Single-cell transcriptome landscape of Macs/Monos

Macs/Monos were independently re-clustered using Harmony-corrected dimensions 1–10 and a clustering resolution of 0.5, yielding 11 initial clusters. Marker-based annotation excluded two residual non-myeloid clusters and consolidated clusters 1/2 and 4/9 on the basis of overlapping defining-marker profiles, resulting in seven Macs/Monos for downstream analyses (**Supplementary Table S1**).

### 2.4 DEG, marker gene and active subgroups identification

Cluster identities were assigned manually rather than by automated reference mapping. Markers were identified with FindAllMarkers in Seurat v5.3.0 under R v4.4.1. For major cell populations, positive markers were identified using only.pos = TRUE and logfc.threshold = 0.25. For independently re-clustered macrophage/monocyte populations, markers were identified using only.pos = TRUE, min.pct = 0.25, and logfc.threshold = 0.5. The five markers with the highest average log2 fold change per cluster were visualized and used to cross-check annotations. Cell identities were based on established lineage-marker combinations and cross-checked against patterns reported in the original GSE233850 publication (19).

### 2.5 Functional enrichment analysis

Functional enrichment was performed with clusterProfiler. Gene Ontology (GO) biological process and KEGG pathway analyses were conducted on differentially expressed genes (DEGs) using the Mus musculus database org.Mm.eg.db. Gene set enrichment analysis (GSEA) ranked all genes by average log2 fold change and evaluated immunometabolic signatures, including oxidative phosphorylation and JAK-STAT, retrieved through msigdbr. Benjamini-Hochberg-adjusted P values were calculated for each gene set. Because GSE233850 contained only one pooled library per condition, these values were not interpreted as biological-replicate-level evidence of significant pathway differences. Results were visualized with enrichplot and ggplot2 (25, 26).

### 2.6 Pseudotime inference and graph-associated gene analysis

To explore putative transcriptional-state relationships among macrophage and monocyte subsets, we performed trajectory inference using Monocle 3 v1.0.0 (27). The selected root imposed directionality on pseudotime ordering and did not establish developmental origin (28). The Seurat object containing seven annotated subtypes was converted to a cell_data_set object and processed using preprocess_cds. No additional alignment was applied because orig.ident represented biological condition rather than an independent technical batch. We then used reduce_dimension and cluster_cells for dimensionality reduction and low-resolution clustering, respectively, and learn_graph to construct a principal trajectory graph. Based on biological context and marker-gene expression, Classical_Inflam_Mono cells were selected as the trajectory root. Cells were ordered along the learned trajectory using order_cells.

Trajectory-associated genes were identified using graph_test with neighbor_graph = “principal_graph”. This function assesses spatial autocorrelation in gene expression among neighboring cells along the learned graph using Moran’s I statistic. Genes with Monocle 3-reported q < 0.001 were retained for exploratory visualization using plot_genes_in_pseudotime. Because each condition comprised a single pooled library, these q values arose from cell-level graph-based analyses. They were not interpreted as evidence of significant differences between biological conditions. The inferred trajectory was likewise treated as a descriptive representation of transcriptional-state relationships, not as direct evidence of lineage progression.

### 2.7 Cell-cell communication analysis

Intercellular communication was inferred with CellChat v1.0.0 using known ligand-receptor pairs to estimate interaction patterns and communication strength across cell types (29, 30). Because each condition comprised a single pooled library, CellChat outputs were interpreted descriptively and were not used for biological-replicate-level comparisons between conditions.

### 2.8 Differential expression analysis of bulk RNA-seq data

To evaluate infection-responsive transcriptional changes in an independent macrophage model, we retrieved bulk RNA-seq dataset GSE283617 (platform GPL34290) from GEO. To match the mouse scRNA-seq discovery dataset, we retained C57BL/6J bone marrow-derived macrophages (BMDMs) exposed to *Bb* and matched media-treated controls. The final comparison included three Bb-stimulated and three control BMDM samples. Media-treated BMDMs served as unstimulated *in vitro* references under matched culture conditions. Gene-level TPM values were transformed as log2(TPM + 1) and subsequently quantile normalized using normalizeBetweenArrays(method = “quantile”) from the limma package. Differential expression was assessed using linear modeling with empirical Bayes moderation. *P* values were adjusted using the Benjamini–Hochberg method, and differentially expressed genes were defined by an adjusted *P* value < 0.05 and an absolute log2 fold change > 1.5. Upregulated and downregulated DEGs were analyzed separately. We intersected BMDM infection-responsive DEGs with genes showing concordant directional changes in ankle-joint Mon/Mac populations from GSE233850. The significance of each overlap was evaluated by Fisher’s exact test using the common detectable gene universe across datasets.

### 2.9 PPI network construction and consensus hub-gene prioritization

DEGs from GSE283617 were intersected with monocyte/macrophage DEGs from GSE233850. The 121 shared genes were submitted to STRING using Mus musculus (taxonomy ID 10090) and a minimum interaction score of 0.400. After removing self-loops and duplicate edges, interactions were converted to an undirected graph and visualized in Cytoscape using a force-directed layout. Node size and color represented degree.

CytoHubba in Cytoscape was used to rank hub genes by five topological measures: Degree, Bottleneck, EcCentricity, maximum neighborhood component (MNC), and maximal clique centrality (MCC). The top 10 genes from each measure were compared in a five-set Venn diagram. Genes ranked among the top 10 by all five measures were designated consensus hub genes.

### 2.10 Quantitative reverse transcription PCR

RAW264.7 cells (Shanghai Zhongqiao Xinzhou Biotechnology) were infected with Bb at MOI = 1 or treated with PBS for 6 h. Total RNA was extracted with the MolPure^®^ Kit and reverse-transcribed using Hifair^®^ III SuperMix (Yeasen Biotechnology).

qRT-PCR was performed on a Gentier 96R System (Tian Long) using Hieff Unicon^®^ qPCR SYBR Master Mix. Cycling comprised 95 °C for 2 min, followed by 40 cycles at 95 °C for 1 s and 60 °C for 30 s. Relative expression was calculated using the 2^(-ΔΔCT) method and normalized to *Gapdh*. Primers (Tsingke Biotechnology) were as follows: *Msr1*: Fwd-CTGTGGAGTGTGGGTGACTC, Rev-CAGCTGGGATTGGGGTCATT; *Gapdh*: Fwd-TGTTGCCATCAATGACCCCT, Rev-TCGCCCCACTTGATTTTGGA.

### 2.11 Expression comparison and exploratory ROC analysis

Normalized expression matrices for GSE283617 and GSE63085 were analyzed in R. Duplicate gene entries were collapsed using avereps. Values were log2-transformed when required and normalized between arrays using normalizeBetweenArrays. For GSE283617, three control BMDM samples were compared with three Bb-stimulated BMDM samples. For GSE63085, we analyzed 28 available acute pretreatment (V1) PBMC samples and 13 healthy-control samples. Samples from post-treatment longitudinal visits were excluded. Gene-wise differences were estimated using linear models and empirical Bayes moderation in limma with lmFit and eBayes. P values were adjusted using the Benjamini-Hochberg method.

ROC curves were generated separately for Mrc1/Msr1 in GSE283617 and MRC1/MSR1 in GSE63085 using pROC (31). AUCs and 95% confidence intervals were estimated by bootstrap resampling. Because these were univariable, in-sample analyses, particularly for the six-sample GSE283617 dataset, the results were treated as exploratory estimates of apparent discrimination. They quantify apparent in-sample discrimination and should not be interpreted as validated diagnostic performance (32).

### 2.12 Reverse network pharmacology and molecular docking analysis

We constructed a source-compound-gene network using a reverse-screening strategy. Curated chemical-gene interaction records were retrieved from the Comparative Toxicogenomics Database (CTD; https://ctdbase.org/) to identify compounds previously associated with MSR1. Identified compounds were then mapped to traditional Chinese medicine (TCM) materia medica and natural sources. Enrichment analysis with clusterProfiler identified sources enriched for these bioactive compounds. The association network was visualized using igraph. Molecular docking then compared predicted poses and relative scores within the selected MSR1 SRCR-domain pocket. Ligand 3D structures were retrieved from PubChem, optimized and energy-minimized in Chem3D, and converted to PDBQT format using AutoDockTools 1.5.7. The MSR1 crystal structure (PDB ID 7DPX) was retrieved from the RCSB Protein Data Bank. In AutoDockTools 1.5.7, water molecules were removed, polar hydrogens and Gasteiger charges were added, and non-polar hydrogens were merged before PDBQT export. A grid box centered on the SRCR-domain pocket was specified in the docking configuration. Docking was performed with AutoDock Vina. The lowest-energy pose for each ligand was retained and visualized in PyMOL 2.5. Docking scores supported within-protocol computational prioritization only and did not establish direct MSR1 binding, affinity, or receptor modulation (33).

### 2.13 *In silico* virtual knockout analysis of *Msr1* and pathway analysis

Potential regulatory functions of Msr1 were evaluated using scTenifoldKnk (34). Virtual knockout analysis used cells from the LA library only. Gene regulatory networks were constructed from 10 independently sampled networks (nc_nNet = 10), with 500 cells randomly sampled per network (nc_nCells = 500). A random seed of 12345 was used for reproducibility. Msr1 was virtually knocked out, and reference and perturbed networks were compared by manifold alignment. Genes with Benjamini-Hochberg-adjusted P < 0.05 were considered significantly differentially regulated. The 20 genes with the largest regulatory perturbations were analyzed for KEGG enrichment using clusterProfiler, with adjusted P < 0.05 indicating statistical significance.

### 2.14 Statistical analysis

Statistical analyses and visualizations were performed using R 4.4.1 and GraphPad Prism 9.0.2. Two-group comparisons used an unpaired Student’s t-test or Mann-Whitney U-test according to normality. For analyses with adequate biological replication, significance was defined as *P* < 0.05 for bench experiments and FDR < 0.05 after Benjamini-Hochberg adjustment for omics datasets. In GSE233850, ankle tissues from 10 mice were pooled into one scRNA-seq library per condition. The library, not the individual cell, was the biological experimental unit. Between-library differences in cell proportions, expression patterns, trajectory features, enrichment profiles, and inferred communication metrics were therefore treated as descriptive and hypothesis-generating. Cell-level *P* or q values were not interpreted as biological-replicate-level evidence.

## 3 Results

### 3.1 Single-cell transcriptome of LA

We analyzed two pooled libraries from GSE233850: one uninfected control library and one library collected 2 weeks after *Bb* infection. After quality control, 16,841 cells were retained. Normalization, PCA using the first 14 dimensions, and UMAP visualization resolved 20 clusters (**Figure 2A**). Manual annotation based on established lineage markers identified nine major cell populations (**Figure 2B**). **Figure 2C** shows the single-cell transcriptomic atlas across the control and LA libraries. Immune populations included Macs/Monos (3,294 cells, 19.56%; Csf1r, C1qa, Adgre1, and Syngr1) and Neutrophils (1,430 cells, 8.49%; S100a9, S100a8, and Csf3r). T/NK Cells comprised 861 cells (5.11%; Ccl5, Nkg7, Cd3d, and Cd3e). B Cells comprised 1,954 cells (11.60%; Cd79a, Cd79b, Ms4a1, and Ighm). DC Cells comprised 1,567 cells (9.30%; H2-Eb1, Cd1c, and Clec4a3). Endothelial Cells comprised 571 cells (3.39%; Cldn5, Pecam1, and Cdh5). Fibroblasts comprised 5,518 cells (32.77%; Col1a1, Col3a1, and Dcn). Synoviocytes comprised 435 cells (2.58%; Prg4, Htra4, and Anxa8). Cycling Cells accounted for 1,211 cells (7.19%; Mki67, Top2a, and Pclaf). **Figure 2E** shows markers for each population. **Figures 2D, F** descriptively compare the pooled libraries. The LA library contained higher observed percentages of Fibroblasts, Cycling Cells, Endothelial Cells, and Synoviocytes, but lower percentages of Macs/Monos, B Cells, T/NK Cells, Neutrophils, and DC Cells.

**Figure 2:**
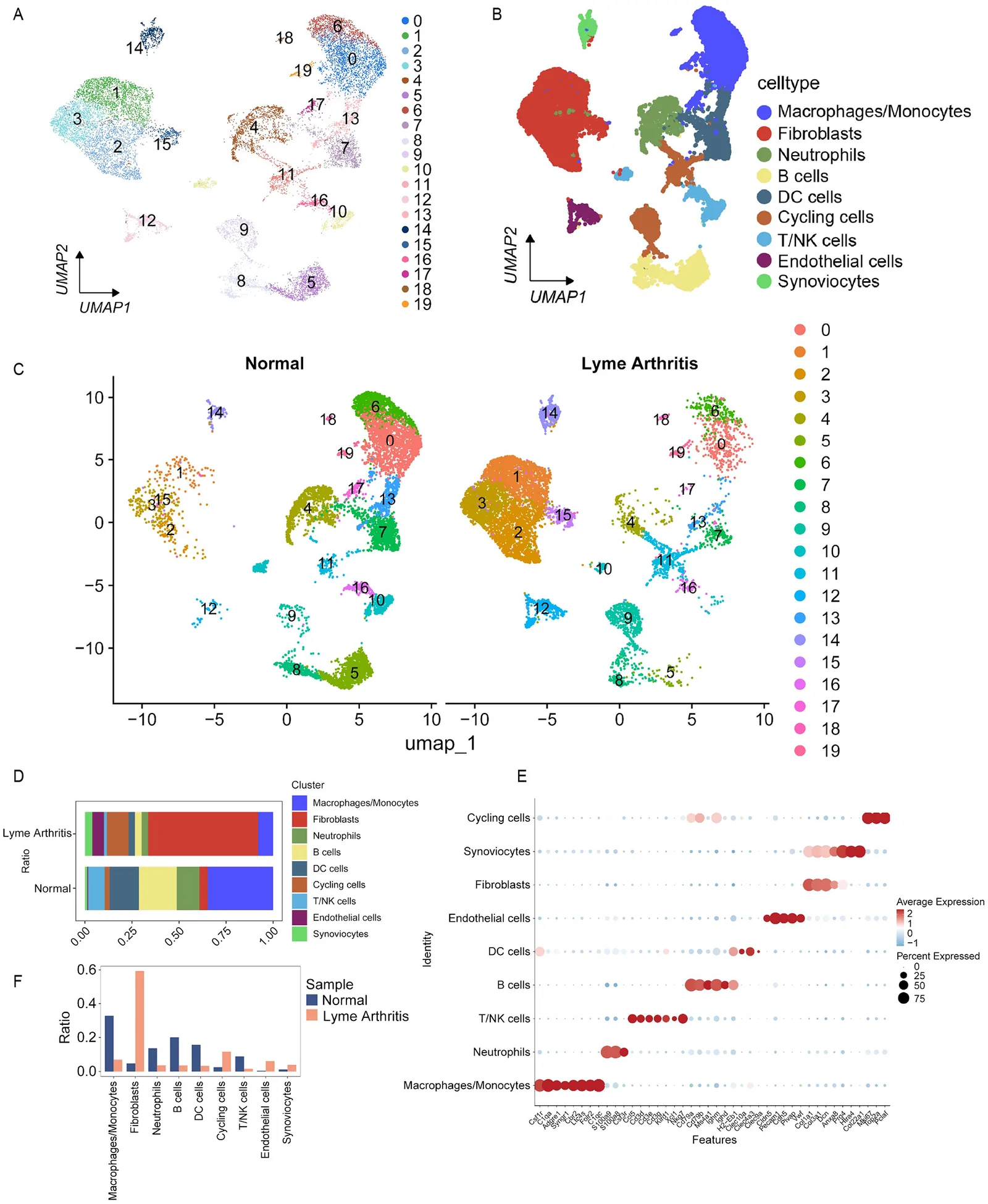
Single-cell atlas of normal and Lyme arthritis C57BL/6 mouse samples. **(A)** UMAP clustering of single cells into 20 clusters; **(B)** Nine major cell populations identified by marker gene expression; **(C)** UMAP comparison between the control and LA libraries; **(D)** Descriptive bar plots of the ratio of different cell types (clusters) between the control and LA libraries; **(E)** Bubble plot of the expression of specific features across different cell types; **(F)** Descriptive bar graph of the ratio of specific cell types between the control and LA libraries.

### 3.2 Single-cell transcriptome landscape of Macs/Monos

Genome-wide relative expression patterns across clusters are shown in **Figure 3A**. The analysis resolved seven marker-defined states (**Figure 3B**). These included LAM_Macro (875 cells; Apoe, Sparc, and S100b), Perivascular_Macro (933 cells; Pf4, Aqp1, and Fxyd2), and Early_activated_Mono (390 cells; Nr4a1, Atf3, and Serpine1). Classical_Inflam_Mono comprised 347 cells (Ccr2, Clec4a1, and Adam8), and Acute_phase_Macro comprised 383 cells (Saa3, Acod1, and Slc13a3). M2_like_Macro comprised 179 cells (Retnla, Itgax, and Flt1), and Proliferating_Macro comprised 107 cells (Cdc20, Mki67, and Top2a). In the descriptive comparison, M2_like_Macro, Early_activated_Mono, Perivascular_Macro, and LAM_Macro had lower observed proportions in the LA library. Proliferating_Macro, Acute_phase_Macro, and Classical_Inflam_Mono had higher observed proportions (**Figure 3C**). These point estimates indicate possible remodeling but cannot establish population-level compositional changes without biological replication. **Figures 3D, E** show the initial and annotated UMAP clusters, respectively. **Figure 3F** shows marker expression supporting the seven state assignments.

**Figure 3:**
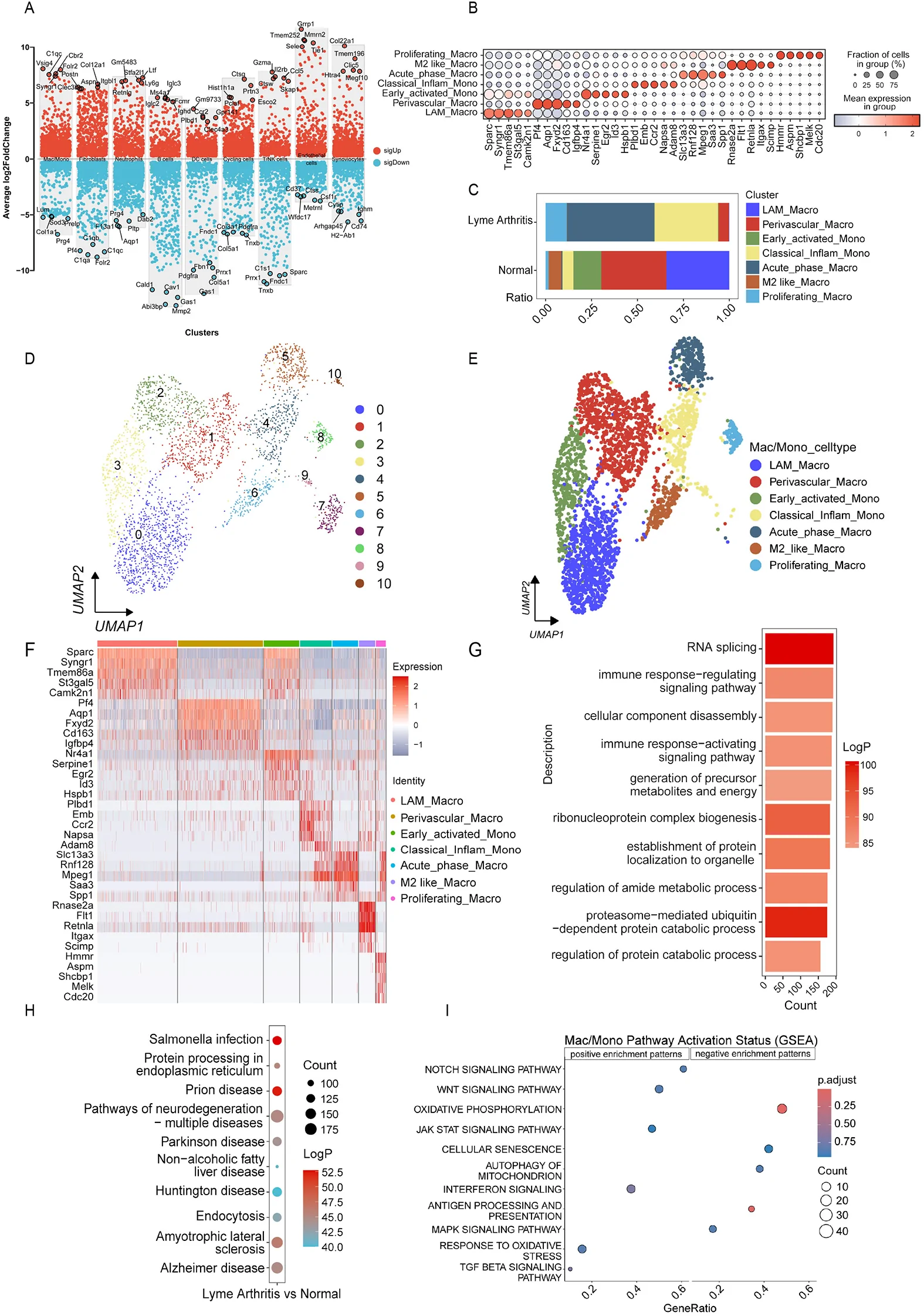
Single-cell transcriptome atlas of Macs/Monos. **(A)** UMAP plot showing differential gene expression across clusters, highlighting relatively upregulated (red) and downregulated (blue) genes; **(B)** Bubble plot showing differential expression patterns of signature genes across 7 Macs/Monos subgroups; **(C)** Descriptive relative Macs/Monos proportions of cell subtypes in the LA versus Control libraries; **(D)** UMAP visualization identifying 11 initial clusters; **(E)** UMAP visualization of 7 transcriptionally distinct monocyte/macrophage clusters; **(F)** Heatmap displaying expression profiles of top marker genes among the Macs/Monos subgroups; **(G)** Exploratory Gene Ontology (GO) functional enrichment analysis of genes showing expression differences between the two pooled libraries; **(H)** Exploratory KEGG pathway enrichment analysis of genes showing expression differences between the two pooled libraries; **(I)** Exploratory GSEA showing relatively positive and negative enrichment patterns of signaling-related gene sets in macrophage/monocyte populations. The plot separates pathways into relatively positive (left) and relatively negative (right) enrichment patterns. Dot size represents gene count, and color indicates the adjusted p-value (p.adjust).

### 3.3 Macs/Monos -related enrichment analysis

We performed GO, KEGG, and GSEA enrichment analyses of macrophage/monocyte DEGs. GO analysis indicated over-representation of RNA splicing, immune-response regulation, cellular-component disassembly, and generation of precursor metabolites and energy (**Figure 3G**). KEGG analysis indicated over-representation of protein processing in the endoplasmic reticulum and Salmonella infection gene sets (**Figure 3H**). Exploratory GSEA showed relatively positive enrichment of NOTCH, WNT, JAK-STAT, and interferon-related gene sets. Oxidative phosphorylation, mitophagy, and antigen-processing and presentation gene sets showed relatively negative enrichment (**Figure 3I**).

### 3.4 Exploratory pseudotime analysis

Monocle 3 was used to infer a principal graph across macrophage/monocyte states. Classical_Inflam_Mono was specified as the root and occupied a low-pseudotime region (**Figure 4B**). Annotated states occupied different positions along the graph (**Figures 4B, C**). LAM_Macro, Perivascular_Macro, and M2_like_Macro occurred mainly in higher-pseudotime regions. These patterns represent putative transcriptional-state relationships, not temporal differentiation or lineage progression. **Figure 4D** shows 10 graph-associated genes across inferred pseudotime. mt-Co1 was highest in Acute_phase_Macro and decreased across pseudotime. Rnase2a, H2-Aa, H2-Eb1, Cd74, Sparc, and Timp2 were more highly expressed at intermediate-to-high pseudotime positions, including regions enriched for M2_like_Macro. Cox8a, Cst3, and Gm42418 remained expressed in regions containing LAM_Macro, where Sparc and Timp2 were also detected. These descriptive observations require validation using lineage-resolved or longitudinal data.

**Figure 4:**
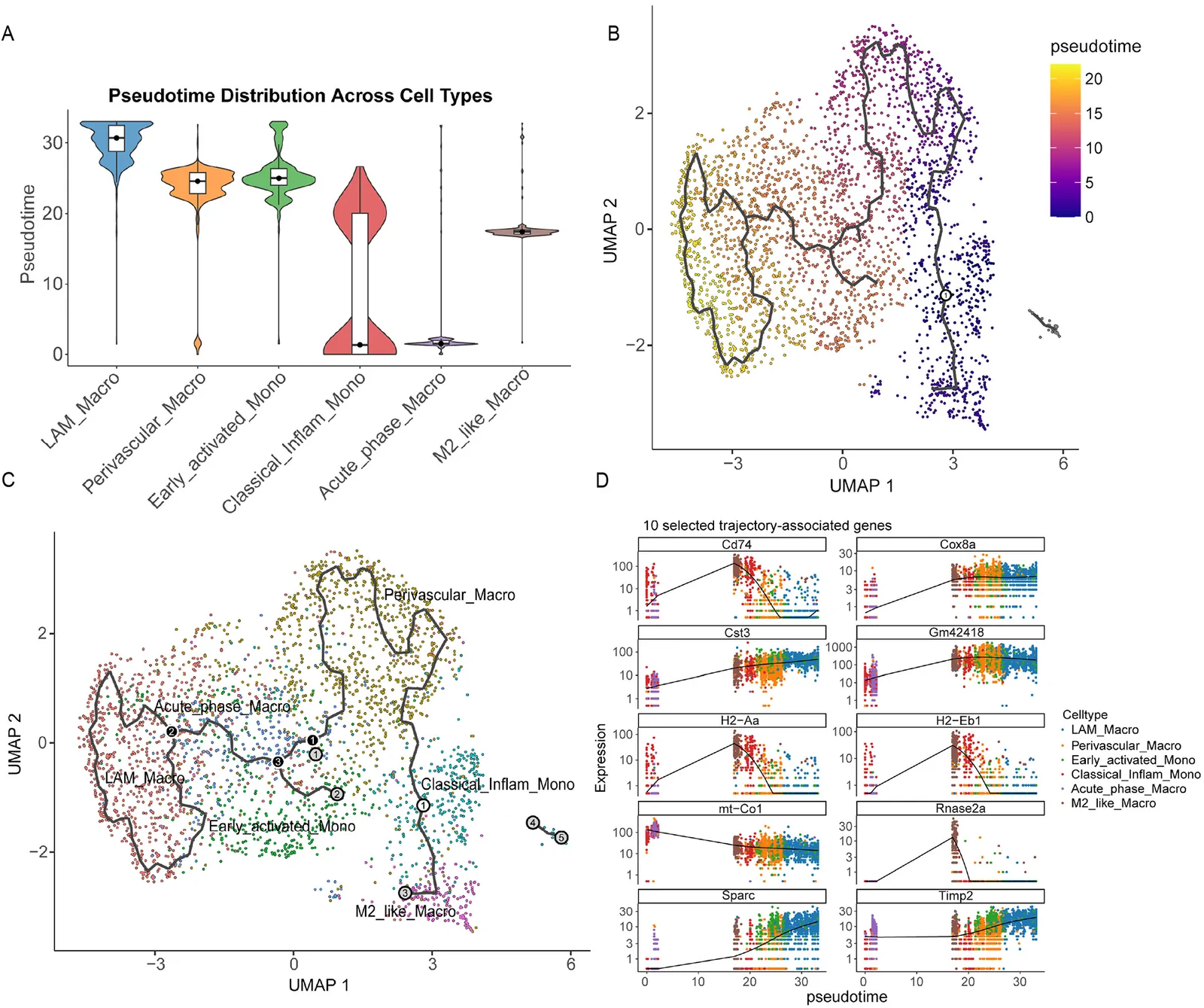
Exploratory pseudotime analysis and graph-associated gene-expression patterns in macrophage/monocyte states. **(A)** Distribution of inferred pseudotime values across the annotated states. Violin plots show the relative positions of individual cells along the inferred pseudotime axis. **(B)** UMAP visualization of cells colored by pseudotime. The dark-purple-to-yellow gradient represents lower-to-higher pseudotime values. The low-pseudotime region overlaps the Classical_Inflam_Mono root area. **(C)** Distribution of annotated states along the learned principal graph. Numbered circles denote graph nodes; gray-filled circles indicate endpoints, white-filled circles indicate branch points, and black-filled circles mark selected internal nodes. The root selected for cell ordering lies within the Classical_Inflam_Mono-associated region and corresponds to the low-pseudotime end in panel **(B, D)** Expression patterns of 10 selected graph-associated genes along inferred pseudotime. Genes were identified using Monocle 3 graph_test with neighbor_graph = “principal_graph” and q < 0.001. Each point represents one cell and is colored by its annotated state; black curves indicate fitted expression trends. The inferred graph and gene-expression patterns are exploratory and do not establish temporal lineage progression or statistically validated differences between biological conditions.

### 3.5 Cell-to-cell communication between macrophage/monocyte and other cells

Cell-cell communication analysis inferred fewer interactions in the LA library than in the control library (641 versus 1,545). Aggregate interaction strength was also lower (16.2 versus 26.75; **Figure 5A**). Sender-receiver patterns differed descriptively. Classical_Inflam_Mono was the main signal source in the control library. Acute_phase_Macro was the main source in the LA library, and Fibroblasts were the main receivers. Acute_phase_Macro and Fibroblasts remained prominent inferred communication hubs (**Figures 5B, C**). These estimates describe two pooled libraries and do not provide replicate-level evidence of a population difference.

**Figure 5:**
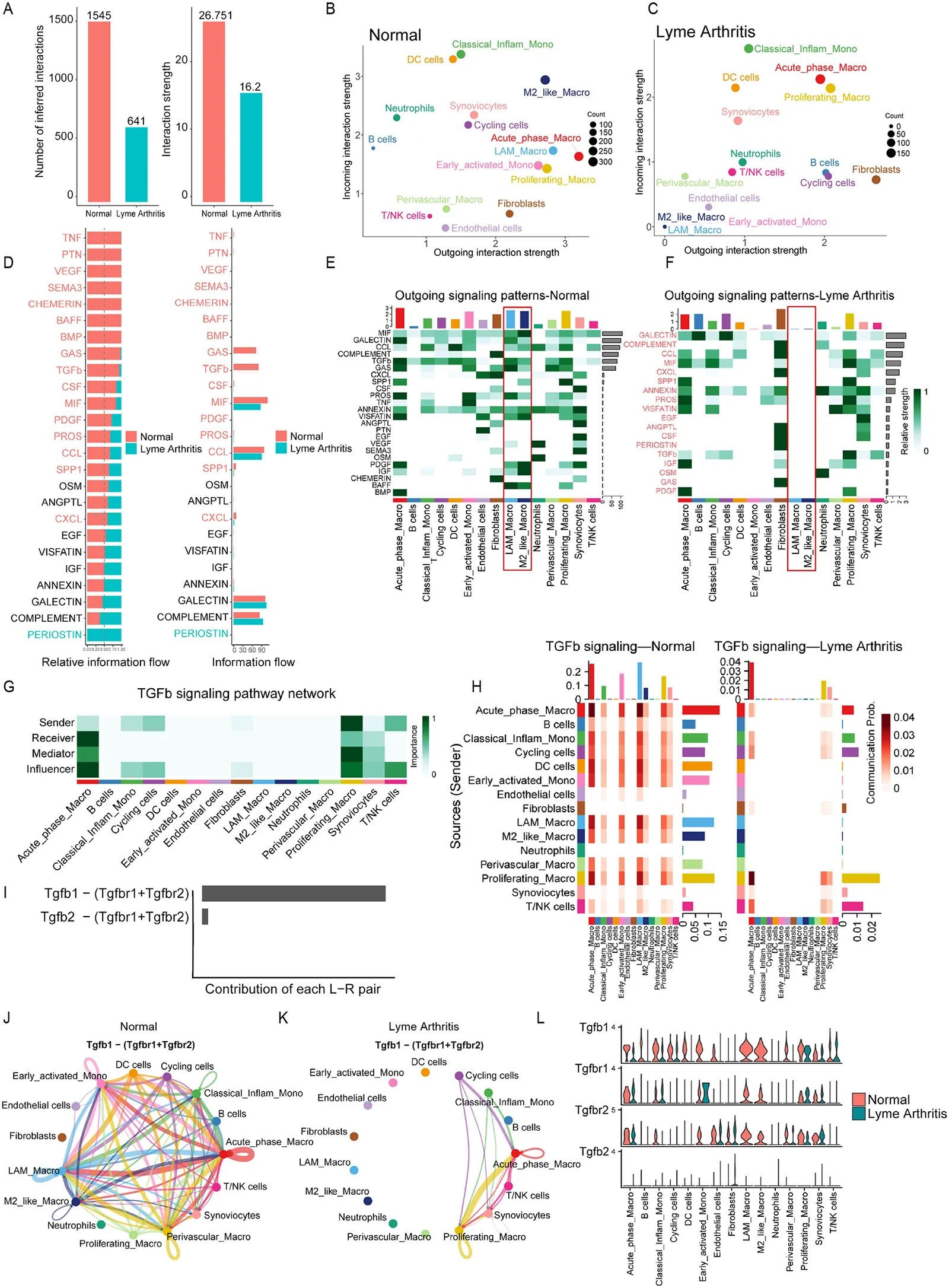
Descriptive patterns of inferred cell–cell communication in the two pooled libraries. **(A)** Descriptive comparison of inferred cell communication networks between the control and LA libraries, assessing the total number of inferred interactions and interaction strength; **(B, C)** Scatter plots summarizing the inferred outgoing and incoming interaction strengths of each cell population in the control and LA libraries, respectively; **(D)** Descriptive comparison of relative information flow for each signaling pathway between control (red) and LA libraries (blue); **(E, F)** Heatmaps characterizing the outgoing signaling patterns of different cell types in Normal **(E)** and Lyme Arthritis **(F)** libraries; **(G)** Network centrality analysis identifying the functional roles (Sender, Receiver, Mediator, and Influencer) of cell types involved in the TGF-β signaling pathway; **(H)** Descriptive comparison of inferred communication probabilities for TGF-β signaling interactions across cell types in both libraries; **(I)** Contribution analysis of key ligand-receptor pairs to the TGF-β signaling pathway. The Tgfb1-(Tgfbr1+Tgfbr2) pair shows the highest contribution; **(J, K)** Chord diagrams visualizing the intercellular communication network of the Tgfb1-(Tgfbr1+Tgfbr2) pair in Normal **(J)** and Lyme Arthritis **(K)** libraries. Edge color represents the source cell type, and edge width represents the interaction strength; **(L)** Violin plots showing the expression levels of TGF-β signaling ligands and receptors across different cell types in Normal and Lyme Arthritis libraries.

Descriptive pathway analysis indicated lower inferred GAS and TGF-β signaling in the LA library, whereas COMPLEMENT and GALECTIN signaling appeared higher (**Figure 5D**). In the control library, Early_activated_Mono, LAM_Macro, and M2_like_Macro showed inferred outgoing CCL, GAS, BAFF, MIF, VISFATIN, COMPLEMENT, GALECTIN, and TGF-β signals. Their inferred outgoing signaling was lower in the LA library (**Figures 5E, F**). This pattern requires validation in biologically replicated datasets.

Given the role of TGF-β in tissue repair and fibrosis, we examined this pathway further. Network centrality analysis identified Proliferating_Macro as a prominent inferred sender and Acute_phase_Macro as a prominent inferred receiver of TGF-β signals (**Figure 5G**). Tgfb1-(Tgfbr1+Tgfbr2) contributed more to the inferred TGF-β network than Tgfb2 interactions (**Figure 5I**). **Figure 5H** was consistent with lower inferred regulatory TGF-β connectivity in the LA library. Chord diagrams similarly showed a denser inferred Tgfb1-mediated network in the control library and a sparser, weaker network in the LA library (**Figures 5J, K**). These observations describe the two pooled libraries and do not establish a replicate-level difference. Tgfb1, Tgfbr1, and Tgfbr2 also showed lower observed expression in the LA library (**Figure 5L**).

### 3.6 Transcriptomic profiling, candidate identification and functional enrichment analysis

To determine whether ankle-joint Mon/Mac infection-responsive genes were also detected in an independent *in vitro* macrophage model, we analyzed C57BL/6J BMDMs from GSE283617. *Bb*-stimulated BMDMs were compared with media-treated controls (**Figures 6A, B**). This cross-context concordance analysis was not used to define macrophage identity genes. Upregulated and downregulated genes were intersected separately, identifying 63 concordantly upregulated and 58 concordantly downregulated genes (**Figure 6C**). Fisher’s exact/hypergeometric testing showed enrichment relative to the common detectable gene background. The upregulated overlap was 63 (OR = 1.99, *P* = 2.60 × 10^-6^), and the downregulated overlap was 58 (OR = 2.06, *P* = 2.64 × 10^-6^).

**Figure 6:**
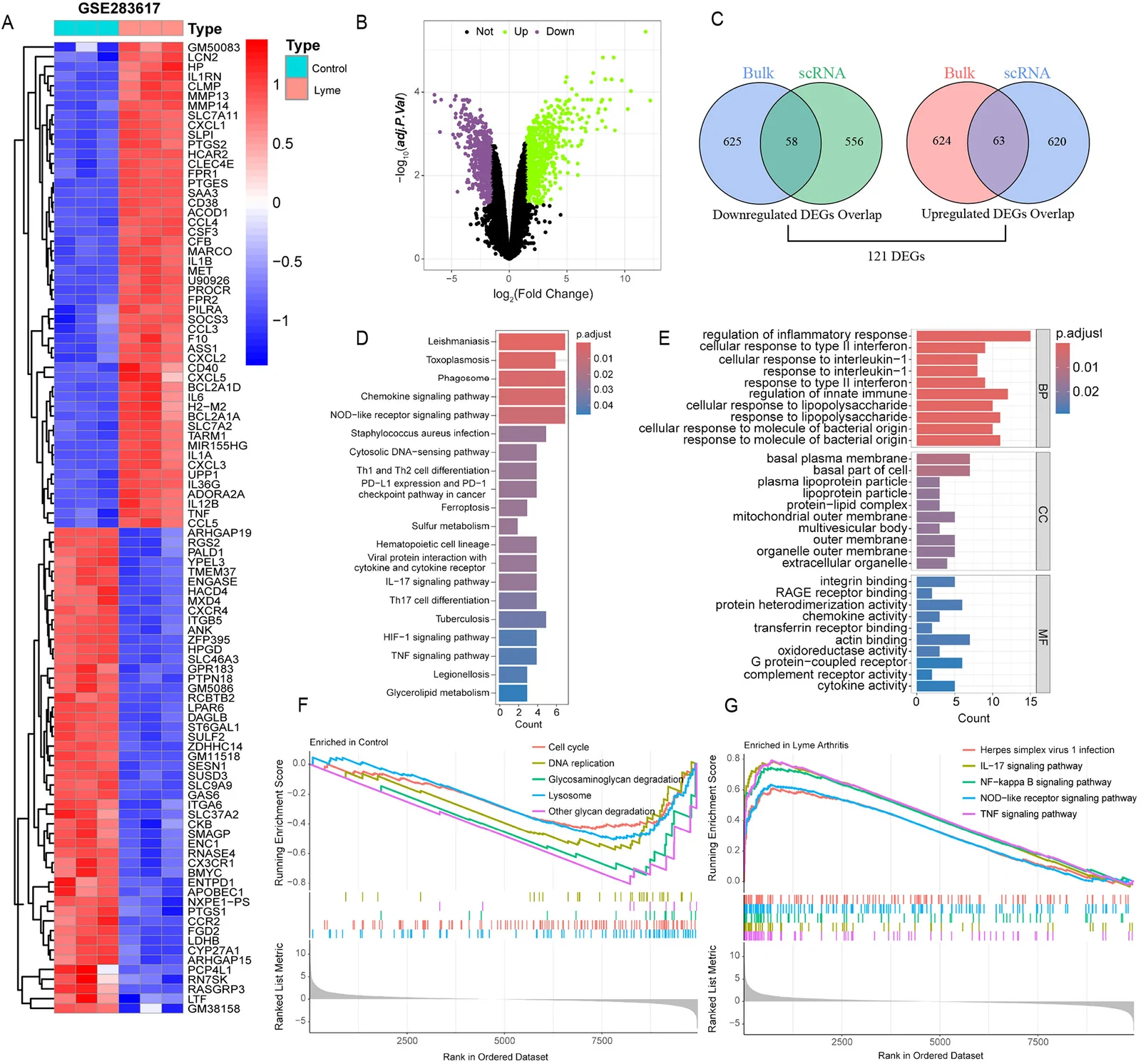
Identification and functional characterization of shared infection-responsive genes. **(A)** Heatmap showing differentially expressed genes (DEGs) between media-treated control and *Bb*-stimulated C57BL/6J bone marrow-derived macrophage (BMDM) samples in GSE283617; Red and blue indicate relatively high and low expression, respectively. **(B)** Volcano plot of DEGs in GSE283617; green, purple, and black points indicate significantly upregulated, significantly downregulated, and non-significant genes, respectively. **(C)** Venn diagrams showing directional overlap between BMDM DEGs from GSE283617 (bulk RNA-seq) and ankle-joint Mon/Mac genes from GSE233850 (scRNA-seq), with 58 concordantly downregulated and 63 concordantly upregulated genes. **(D)** KEGG pathway enrichment analysis of the shared DEGs. **(E)** Gene Ontology enrichment analysis of the shared DEGs, grouped into biological process (BP), cellular component (CC), and molecular function (MF). **(F, G)** Gene set enrichment analysis showing pathways relatively enriched in the control **(F)** and *Bb*-stimulated BMDM **(G)** groups.

KEGG and GO analyses characterized the 121 shared DEGs (**Figures 6D, E**). KEGG terms included Leishmaniasis, Toxoplasmosis, Chemokine signaling, and NOD-like receptor signaling. GO biological process terms included regulation of inflammatory responses and cellular responses to type I interferon and lipopolysaccharides. Cellular component terms included the basal plasma membrane and lipoprotein particles, whereas molecular function terms included integrin binding and RAGE receptor binding. GSEA further identified pathways enriched in the control group, including Cell cycle, DNA replication, and Lysosome (**Figure 6F**). In the LA group, enriched pathways included IL-17, NF-kappa B, NOD-like receptor, and TNF signaling (**Figure 6G**).

### 3.7 Multi-metric topological analysis of the PPI network identifies Mrc1 and Msr1 as consensus hub genes

The STRING network derived from 121 intersecting genes contained 72 connected nodes and 229 interactions (**Figure 7A**). Mrc1 had the highest degree (22), followed by Apoe (20), Ccr5 (18), Igf1 (17), and C1qa and Cybb (16 each). Msr1 had 15 interactions within the same highly connected macrophage-associated module. Across the five top-10 lists, eight genes appeared once, one appeared twice, two appeared three times, six appeared four times, and two appeared in all five lists (**Figure 7B**). Only Mrc1 and Msr1 ranked among the top 10 by Degree, Bottleneck, EcCentricity, MNC, and MCC, supporting their prioritization as consensus topological hubs. Because **Figures 7A, B** were derived from the same PPI graph, they provide complementary but not independent topological evidence.

**Figure 7:**
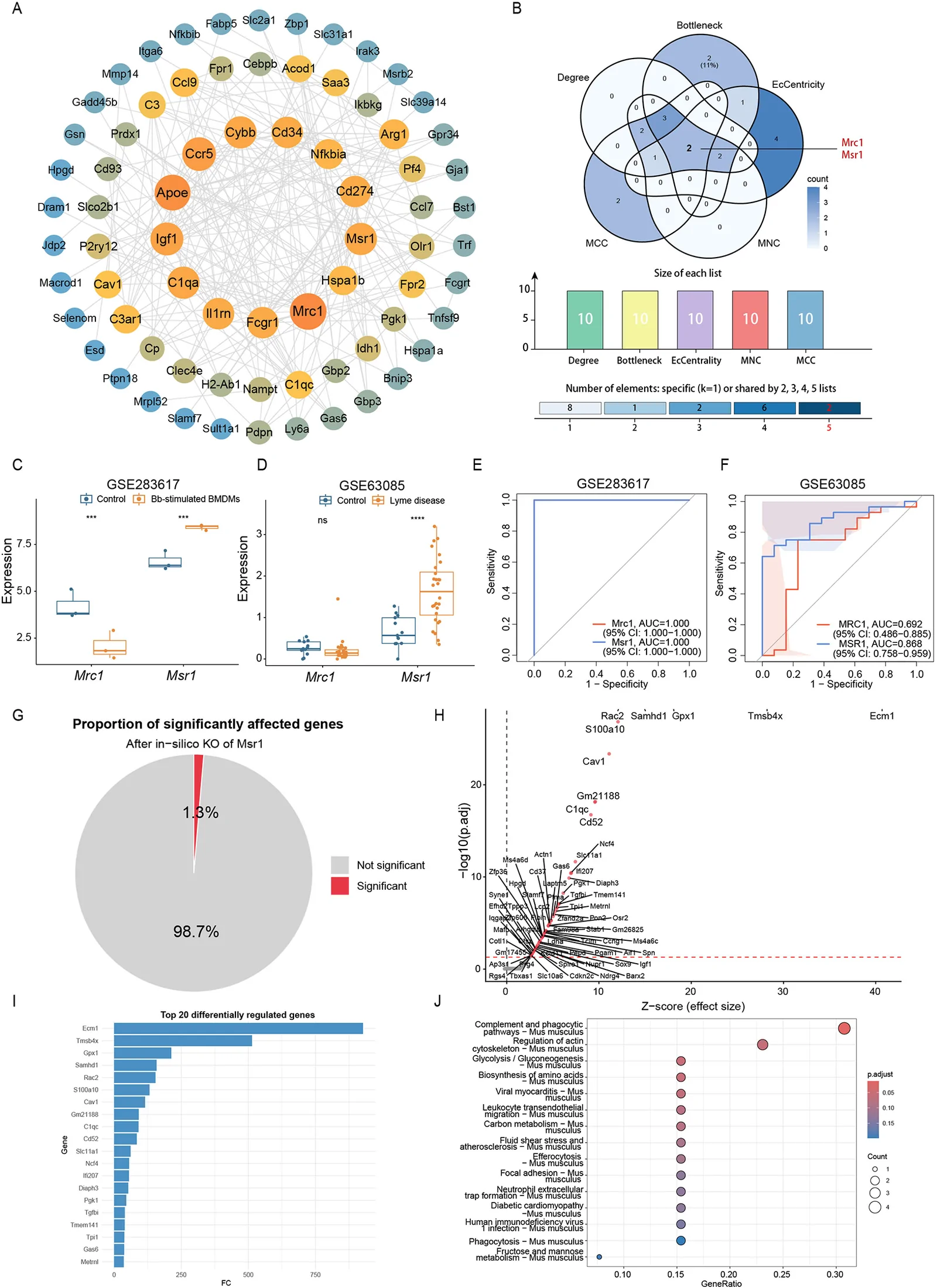
Network prioritization of Mrc1/Msr1 and in silico analysis of Msr1 perturbations. **(A)** PPI network (72 nodes, 229 edges) built from genes shared between *Bb*-stimulated mouse BMDM DEGs (GSE283617) and monocyte/macrophage DEGs (GSE233850). Node size/color reflect degree (orange=high, green=mid, blue=low). **(B)** Venn diagram comparing top-10 genes ranked by Degree, Bottleneck, EcCentricity, MNC, and MCC; Mrc1 and Msr1 alone ranked top-10 across all five measures. **(C)** Mrc1 and Msr1 expression in control and *Bb*-stimulated mouse BMDMs from GSE283617 (n = 3 per condition). **(D)** MRC1 and MSR1 expression in healthy controls (n = 13) and patients with acute pretreatment Lyme disease (n = 28) from GSE63085. **(E, F)** ROC curves: AUC = 1.000 for both genes in the GSE283617 **(E)**; AUC = 0.692 (MRC1) and 0.868 (MSR1) in the GSE63085 **(F)**. **(G–I)** In silico *Msr1* knockout (scTenifoldKnk) in murine scRNA-seq data(GSE233850): 67/4,998 genes (1.3%) significantly perturbed (FDR<0.05) **(G)**; Z-score vs. −log10(FDR) plotted per gene **(H)**; top 20 perturbed genes shown **(I)**. **(J)** KEGG analysis of these 20 genes (19 mapped, 13 annotated); top 15 pathways shown, dot size=gene count, color=adjusted *P* value. AUC, area under curve; FDR, false-discovery rate; KEGG, Kyoto Encyclopedia of Genes and Genomes; MNC, maximum neighborhood component; MCC, maximal clique centrality; ns, not significant; ***P < 0.001; ****P < 0.0001.

### 3.8 Cross-dataset evaluation of MRC1 and MSR1 expression in *Bb*–exposed macrophages and human Lyme disease

In GSE283617, *Bb* stimulation was associated with lower Mrc1 expression (*P* = 0.004) and higher Msr1 expression (*P* = 0.002) than in control BMDMs (**Figure 7C**). In the independent human GSE63085 cohort, MRC1 did not differ between Lyme disease and control groups (*P* = 0.412). MSR1 was higher in Lyme disease (*P* = 1.83 × 10^-4^; **Figure 7D**). Exploratory ROC analysis quantified within-dataset discrimination between source-defined groups. Both Mrc1 and Msr1 yielded apparent AUCs of 1.000 (95% CI, 1.0–1.0) in GSE283617 (**Figure 7E**). In GSE63085, the AUC was 0.692 (95% CI, 0.486–0.885) for MRC1 and 0.868 (95% CI, 0.758–0.959) for MSR1 (**Figure 7F**). MSR1 therefore showed a more consistent cross-dataset signal than MRC1.

### 3.9 Virtual Msr1 knockout identifies a restricted set of network-sensitive genes and KEGG analysis

Virtual Msr1 knockout significantly altered the inferred regulatory state of 67 of 4,998 tested genes (1.3%; FDR < 0.05). The remaining 4,931 genes (98.7%) were not significant (**Figure 7G**). Significant perturbations were concentrated at large positive standardized manifold-distance scores (**Figure 7H**). The largest statistics were observed for Ecm1, Tmsb4x, Gpx1, Samhd1, Rac2, S100a10, Cav1, Gm21188, C1qc, Cd52, Slc11a1, Ncf4, Ifi207, Diaph3, Pgk1, Tgfbi, Tmem141, Tpi1, Gas6, and Metrnl (**Figure 7I**).

KEGG analysis of the top 20 perturbed genes returned 63 annotated terms, and the 15 highest-ranked terms are shown in **Figure 7J**. Only Complement and phagocytosis remained significant after multiple-testing correction (gene ratio = 4/13; adjusted *P* = 0.013). Other highly ranked terms included regulation of the actin cytoskeleton, glycolysis/gluconeogenesis, amino-acid biosynthesis, leukocyte transendothelial migration, efferocytosis, focal adhesion, and phagocytosis. These terms did not meet FDR < 0.05 (adjusted *P* = 0.055–0.199).

### 3.10 Computational prioritization of MSR1-associated compounds and assessment of Msr1 expression

Intersecting CTD-derived MSR1-associated chemicals with the expanded TCM database identified five compounds linked to MSR1 and 10 enriched materia medica and natural sources (adjusted *P* < 0.05; **Figures 8A, B**). Arsenic trioxide was retained in the database overlap but excluded from docking because its inorganic, speciation-dependent chemistry is not directly comparable with the four organic compounds. In aqueous biological environments, arsenic trioxide generates trivalent arsenic species whose protein interactions may involve thiol coordination or covalent reactivity (35). These processes are not adequately represented by the conventional non-covalent AutoDock Vina workflow used here (36, 37). Docking was therefore restricted to curcumin, demethoxycurcumin, bisdemethoxycurcumin, and resveratrol, which could be evaluated using a consistent ligand-processing and scoring procedure. Predicted docking scores for the human MSR1 SRCR domain were -7.0, -7.0, -6.9, and -5.9 kcal mol^−1, respectively (**Figure 8C**). Curcumin, demethoxycurcumin, and bisdemethoxycurcumin occupied a shared predicted pocket containing Arg378, Val417, Phe418, Phe420, Glu423, Glu428, Cys429, Lys430, and Arg432. Predicted polar contacts included Val417, Glu423, Glu428, and Lys430 (**Figures 8E, F, H**). Resveratrol adopted a partly distinct pose involving Glu416, Arg432, Gln433, Arg437, and surrounding residues (**Figure 8G**). Separately, *Bb* exposure increased Msr1 mRNA expression in RAW264.7 macrophages relative to PBS treatment (*P* = 0.0002; **Figure 8D**). This assay measured the transcriptional response to *Bb* and did not test any candidate compound.

**Figure 8:**
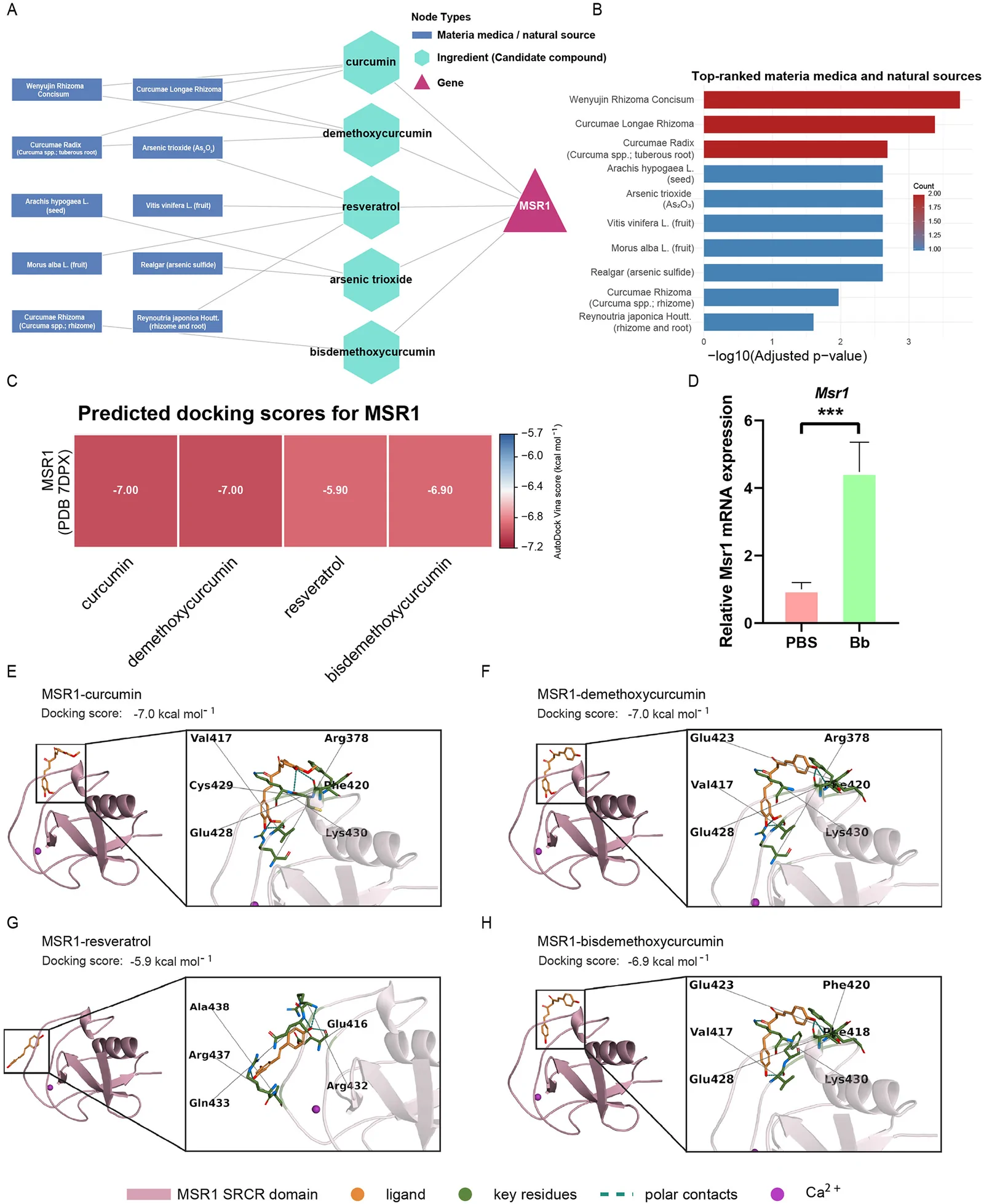
Computational prioritization of MSR1-associated compounds and independent assessment of Msr1 expression. **(A)** MSR1-centered source–compound association network: 10 enriched materia medica and natural sources (blue rectangles), five compounds (cyan hexagons), and MSR1 (magenta triangle); edges denote documented associations. **(B)** Over-representation analysis of the five compounds against source-associated compound sets; bars show the ten enriched materia medica and natural sources ranked by −log10(adjusted P), colored by compound count. **(C)** Best AutoDock Vina scores for curcumin, demethoxycurcumin, resveratrol, and bisdemethoxycurcumin docked to the human MSR1/SCARA1 SRCR domain (PDB ID: 7DPX); more negative scores indicate more favorable poses. **(D)** Relative Msr1 mRNA expression in RAW264.7 cells exposed to *Bb* or PBS. This assay assessed the transcriptional response to Bb exposure and did not test any prioritized compound (mean ± s.d.; n = 3 biological replicates). Statistical significance was assessed using an unpaired two-tailed Student’s t-test, ***P < 0.001. **(E–H)** Predicted poses of curcumin **(E)**, demethoxycurcumin **(F)**, resveratrol **(G)**, and bisdemethoxycurcumin **(H)** in the SRCR domain (best scores −7.0, −7.0, −5.9, −6.9 kcal mol^-1^). Domain, pink cartoon; ligands, orange sticks; pocket residues, green sticks; polar contacts within 3.6 Å, cyan dashes; crystallographic Ca^2+^, magenta sphere. Insets show ligand-contact environments; residues numbered per UniProt P21757.

## 4 Discussion

This study identified coordinated, although exploratory, remodeling of the ankle-joint macrophage/monocyte compartment after *Bb* infection. The infected pooled library showed greater representation of Acute_phase_Macro, Classical_Inflam_Mono, and Proliferating_Macro. It showed lower representation of Early_activated_Mono, M2_like_Macro, LAM_Macro, and Perivascular_Macro. These compositional differences were accompanied by directionally weaker oxidative phosphorylation (OXPHOS) and TGF-β-related programs. By integrating cell-state patterns with immunometabolic and intercellular-signaling features, our analysis extends the original time-course single-cell atlas (19). Together, the findings suggest that peak joint inflammation may reflect an imbalance between inflammatory recruitment or activation and the acquisition or maintenance of reparative macrophage states. This interpretation is more consistent with the observed heterogeneity than a uniform change across the macrophage pool.

The metabolic and subpopulation patterns are most informative when considered together. Enrichment of the broad Gene Ontology term “generation of precursor metabolites and energy” does not itself establish glycolysis. However, directionally lower OXPHOS and mitophagy signatures, an Acod1-high acute-phase state, and relatively expanded inflammatory populations are compatible with altered mitochondrial and glycolytic programs. Such programs may favor rapid ATP production and inflammatory mediator generation over fatty-acid oxidation and electron transport linked to efferocytosis, IL-10, and repair (38–42). The reduced M2_like_Macro, LAM_Macro, and Perivascular_Macro populations may therefore indicate limited lipid-handling and reparative capacity, rather than lower energy output itself. ACOD1 generates itaconate, an inhibitor of succinate dehydrogenase (41, 43). The Acod1-high state could therefore couple antimicrobial activation to an intrinsic metabolic constraint. Collectively, these patterns suggest that the *in vivo* subsets may not be fully represented by a binary M1/M2 framework.

This interpretation accords with Barriales et al. (44), who found that prior *Bb* exposure increased glycolytic capacity and lactate in memory-like macrophages. Glycolysis inhibition also reduced TNF production and inflammation in that model. Conversely, Kerstholt et al. (45) reported lower baseline lactate in human monocytes after *Bb* exposure. This divergence suggests that *Bb*-induced immunometabolism varies with species, cell source, tissue context, and acute versus recall responses. Our two-week joint profile therefore supports a tissue-level association between metabolic state and the balance of inflammatory and reparative subpopulations. However, it does not establish increased glycolytic flux. Extracellular acidification, oxygen consumption, glucose uptake, lactate, and targeted metabolite measurements in sorted joint subsets are required to test the proposed OXPHOS-to-glycolysis transition.

The trajectory and communication analyses suggest a possible connection between macrophage metabolism and joint pathology. Classical_Inflam_Mono was specified as the root for cell ordering. M2_like_Macro, Perivascular_Macro, and LAM_Macro occupied other graph regions with distinct mitochondrial and remodeling features, including Cox8a, Sparc, and Timp2. This topology is consistent with, but does not prove, a transition from recruited inflammatory cells toward specialized tissue states. In parallel, the infected library showed weaker inferred Tgfb1-(Tgfbr1+Tgfbr2) connectivity, particularly between macrophage and stromal compartments. This occurred alongside greater observed representation of fibroblasts and cycling cells. Human postinfectious LA synovium shows strong interferon activity and suppressed tissue-repair programs (7, 46, 47). This disease context supports testing whether myeloid-stromal repair coordination is altered. Nevertheless, TGF-β is context dependent, and local TGF-β1 inhibition altered the Th17/Treg balance without improving experimental joint pathology (48).

MSR1, also known as SR-AI/II or CD204, is a class A scavenger receptor expressed predominantly on macrophages. It is a homotrimeric type II transmembrane glycoprotein with extracellular coiled-coil and collagen-like domains. SR-AI also contains a C-terminal scavenger receptor cysteine-rich (SRCR) domain. In the alternatively spliced SR-AII isoform, the SRCR domain is replaced by a short six-amino-acid C-terminal sequence (49, 50). MSR1 was prioritized because its infection-associated signal was more consistent across datasets and validation levels. Msr1 was upregulated in *Bb*-stimulated BMDMs and RAW264.7 cells, while MSR1 was also elevated in the human dataset. It also emerged as a consensus PPI hub, and virtual knockout mainly affected complement- and phagocytosis-related genes. Class A scavenger receptors participate in non-opsonic bacterial uptake and host defense (51, 52). More directly, MSR1 was detected in a *Bb*-enriched phagosomal fraction and mediated spirochetal binding when ectopically expressed in non-phagocytic CHO cells (53). Msr1 silencing in macrophages increased both spirochetal uptake and TNF production (53). These findings indicate that MSR1 restrains phagocytosis and inflammatory output in this setting, rather than functioning as a conventional uptake receptor. MRC1 appears more closely associated with regulatory and reparative macrophage states. CD206-positive macrophages predominated in murine LA and remained detectable in ex vivo *Bb*-exposed human synovium (8, 54). However, these observations do not establish a receptor-specific antimicrobial role. MRC1 can also be exploited by pneumococci to suppress inflammation and promote bacterial persistence, illustrating its context-dependent function during infection (55). In our study, Mrc1 decreased in BMDMs and remained unchanged in the human dataset. We therefore prioritized MSR1 because of its stronger cross-dataset concordance and the association with phagocytosis and inflammatory output. Its predicted associations with phagocytosis, complement, and metabolism provided additional support. Nevertheless, MSR1 remains a mechanistic candidate that requires functional validation.

The CTD-based source-compound-gene network and docking analyses jointly prioritized curcumin and demethoxycurcumin for experimental follow-up. Both compounds achieved the most favorable predicted docking score (−7.0 kcal mol^-1^) and occupied a similar pocket within the MSR1 SRCR domain. This convergence is compatible with the predicted binding region but does not confirm direct binding. Previous studies provide indirect macrophage-context plausibility because curcumin and demethoxycurcumin can modulate class A scavenger receptor expression and activity in macrophage models (56). Neither compound has been directly evaluated in *Bb* infection or LA. Curcumin enhanced mycobacterial clearance in human macrophages through autophagy-related and NF-κB-associated mechanisms (57). Demethoxycurcumin suppressed MAPK/NF-κB-mediated inflammatory responses and showed antibacterial activity against MRSA (58). These effects may be relevant because *Bb* activates TLR2-, NF-κB-, and MAPK-dependent inflammatory signaling in macrophages (59). However, the nearly comparable score of bisdemethoxycurcumin precludes a clear affinity-based distinction. Curcumin and demethoxycurcumin should therefore be regarded as prioritized candidates requiring direct binding, MSR1-dependent functional, and anti-*Bb* validation.

Several limitations constrain interpretation of these findings. First, GSE233850 contained one pooled control library and one pooled post-infection library, effectively providing one biological replicate per condition. Each library combined ankle tissues from 10 mice. Pooling increased cell yield but did not provide independent biological replication, and individual cells could not serve as biological replicates for statistical inference. Accordingly, differences in cell and subpopulation abundance, gene expression, trajectory-associated features, and inferred cell-cell communication are descriptive estimates from two libraries. Formal between-condition inference was not possible, and these findings should be considered exploratory and hypothesis-generating. By contrast, GSE283617 contained three independent replicates per condition and supported formal differential-expression testing. External datasets and experimental assays were evaluated separately using their respective statistical procedures.

Second, this study relied primarily on mouse and *in vitro* macrophage models and lacked direct single-cell validation in human LA synovial tissue. BMDMs do not reproduce the heterogeneous tissue-resident and monocyte-derived populations or stromal signals present in inflamed ankle joints. Therefore, overlap between GSE283617 and the joint dataset indicates shared infection-responsive transcriptional changes across macrophage-related contexts, rather than a universal macrophage signature. The external human dataset GSE63085 comprised PBMC samples from patients with acute Lyme disease and healthy controls. It therefore provides indirect evidence of reproducible *Bb*-associated MSR1 expression but cannot establish LA specificity, joint-level relevance, or clinical diagnostic performance. These cross-species, cross-tissue, and cross-model differences limit clinical extrapolation. Validation requires well-characterized LA cohorts with joint-derived specimens and paired blood samples.

Mrc1 underwent the same transcriptomic comparisons and exploratory ROC assessment as Msr1, but it was not evaluated by qRT-PCR or functional experiments. Finally, the compound analysis was entirely computational, whereas qRT-PCR assessed only the Msr1 mRNA response to *Bb* exposure. The experiment did not test curcumin, demethoxycurcumin, or any other prioritized compound. CTD associations and docking predictions cannot establish direct MSR1 binding, altered receptor activity, anti-inflammatory effects, or therapeutic efficacy in LA. These hypotheses require biophysical binding assays, MSR1-dependent cellular experiments, compound-treatment studies, and *in vivo* validation.

## 5 Conclusion

In conclusion, our exploratory integrative analysis describes a possible monocyte/macrophage remodeling pattern in LA across the two pooled scRNA-seq libraries. The observed compositional, TGF-β signaling, and metabolic differences should be regarded as hypothesis-generating because the single-cell comparison lacked independent biological replicates. Across the complementary analyses, MSR1 emerged as a candidate infection-responsive gene with potential relevance to LA. Its disease specificity, joint-level relevance, and clinical utility require further validation in larger, biologically replicated studies incorporating joint-derived specimens from patients with clinically confirmed LA.

## Data Availability

Publicly available datasets that support the findings of this study are openly available in the Gene Expression Omnibus (GEO) database (https://www.ncbi.nlm.nih.gov/geo/); GSE233850, GSE283617, GSE63085. Additional information for data re-analysis is available from thelead contact upon request.
